# Opportunities and aspirations: the Korea Disease Control and Prevention Agency’s partnership with the Global Outbreak Alert and Response Network

**DOI:** 10.5365/wpsar.2024.15.5.1093

**Published:** 2025-10-20

**Authors:** Bryan Inho Kim, Sangwoo Tak

**Affiliations:** a Division of Infectious Disease Control, Korea Disease Control and Prevention Agency, Cheongju, Republic of Korea.; b Division of Risk Assessment, Korea Disease Control and Prevention Agency, Cheongju, Republic of Korea.

The Global Outbreak Alert and Response Network (GOARN) is a network of institutions that provides technical support to countries responding to public health emergencies, upon request. (*1*) Since its establishment in 2000, GOARN has steadily expanded to include over 310 partner institutions (*2*) and brings more than two decades of experience in facilitating the rapid deployment of experts to support outbreak response operations worldwide. (*3*)

Republic of Korea has three GOARN partners: the Korea Disease Control and Prevention Agency (KDCA), the Lee Jong Wook Global Medical Center and the National Medical Center. KDCA serves as the country’s national public health authority and holds primary responsibility for coordinating responses to public health emergencies. It has led national technical support during international public health events, including the Middle East respiratory syndrome coronavirus (MERS-CoV) outbreak and, more recently, the COVID-19 pandemic.

While deployment through GOARN has not yet occurred for the three institutions during their 15-year partnership, engagement is increasing, with two offers of assistance submitted in response to the 2022 Sudan virus disease outbreak in Uganda. Despite the absence of deployments through GOARN, KDCA has been actively involved in other network activities, including participating in the GOARN Capacity Strengthening and Training Programme and providing technical assistance to the GOARN Operational Support Team (OST) (*3*) at WHO headquarters in Geneva, Switzerland.

## Participation in the GOARN Capacity Building and Training Programme

The GOARN Capacity Building and Training Programme is a multitiered initiative aimed at enhancing the technical skills of outbreak response experts while preparing them to work effectively in multidisciplinary and culturally diverse teams. It focuses on building the core capacities needed for international responders to adapt and apply their expertise in various outbreak response teams and field settings. (*4*)

In 2018, a public health officer from KDCA participated in the 5-day GOARN Outbreak Response Scenario Training (Tier 2) in Brazzaville, Democratic Republic of the Congo. This training emphasizes team building, problem solving during rapidly evolving outbreak scenarios, stakeholder interviews, data analysis and the development of contextual control strategies. It provides participants with a realistic understanding of the challenges faced by international multidisciplinary outbreak response teams. More recently in 2024, the Orientation to International Outbreak Response with GOARN (Tier 1.5) training was hosted by KDCA in Seoul, with the participation of staff from KDCA and the National Medical Center interested in international outbreak response. The training offered participants a valuable opportunity to engage in real-world scenarios through interactive table-top exercises.

## Technical assistance to the GOARN Operational Support Team

In 2019, KDCA facilitated a temporary assignment of one of its staff members to the GOARN OST. (*5*) The secondment was a temporary transfer of 1 year with the staff member remaining employed by KDCA. This assignment was designed to share expertise, develop skills and address specific organizational needs, including supporting the alert and risk assessment sector in the network. It reflected KDCA’s priority to expand its international collaborative network.

During the assignment, the staff member actively contributed to a range of activities, including supporting the management of alert and risk assessment functions, implementing standard operating procedures such as screening candidates for deployments, and coordinating the GOARN weekly operational call to facilitate information-sharing for ongoing field operations.

## Benefits of GOARN engagement

Participation in the 5-day GOARN Outbreak Response Scenario Training provided the KDCA participant with hands-on experience in managing effective field responses during rapidly evolving and complex public health emergencies. The experience proved invaluable during the COVID-19 pandemic response in Republic of Korea, where the participant applied skills gained in the training, such as conducting rapid risk assessment, coordinating with multidisciplinary teams and applying interventions based on rapid decision-making. The participant also shared their experience and knowledge with KDCA’s newly hired epidemiological investigation officers through the Korean field epidemiology training programme. This facilitated the strengthening of institutional response capacity by transferring practical outbreak response experiences and expertise, encouraging active participation in international public health issues, and mentoring new staff for future public health emergencies.

The secondment of a KDCA staff member to the GOARN OST provided an opportunity to contribute meaningfully to the expansion of the Epidemic Intelligence from Open Sources (EIOS) initiative. EIOS is a key tool for comprehensive event-based surveillance and facilitates real-time information-sharing among WHO Member States and partners. The EIOS platform supports collaboration and coordination among partner organizations involved in monitoring and assessing public health threats.

This collaboration between KDCA and the EIOS core team at WHO headquarters led to the hosting of the 2019 EIOS Global Technical Meeting held from 12–14 November 2019 in Seoul, Republic of Korea. (*6*) The event brought together many GOARN partners and featured active discussions on improving information-sharing and strengthening collaboration across the Network. Hosting the meeting laid the foundation for continued technical cooperation and contributed to the global development and expansion of the EIOS platform. (*7*)

Following the year-long secondment, the staff member was appointed as KDCA’s liaison officer to WHO, with the primary role of advancing institutional collaboration. The secondment yielded lasting and tangible benefits at both individual and institutional levels. Individually, the officer gained valuable international experience and career development opportunities. Institutionally, KDCA strengthened its global networks, enhanced its visibility within the GOARN community, and deepened its engagement with WHO and other partner institutions. These outcomes align with KDCA’s strategic priority to expand its international engagement following the COVID-19 pandemic.

## What next?

Strengthening KDCA's contribution to the GOARN network is vital to the enhancement of global health security and outbreak response capacity. Greater involvement in the GOARN Capacity Building and Training Programme is essential, as it provides experts with opportunities to refine their field skills and prepares them to effectively operate in multisectoral and international response teams. This not only advances KDCA’s ability to respond to global health emergencies but also contributes to building a more robust and coordinated global response framework.

It is essential to establish streamlined administrative procedures, including the development of a roster of qualified experts, to facilitate the deployment of technical experts. This roster would enable the rapid identification and mobilization of suitable candidates, ensuring timely and effective responses to requests for assistance.

Ongoing communications between KDCA and the GOARN OST as well as active participation in GOARN partner engagement activities should be prioritized. These mechanisms offer valuable insights into outbreak response efforts, foster networking and opportunities for collaboration, and enable knowledge-sharing with other partner institutions to enhance collective efforts and facilitate effective field responses.

KDCA has made valuable contributions to GOARN through diverse activities and now has a chance to enhance this collaboration further. The expansion of KDCA’s role in global health, particularly in outbreak field response, is a valuable opportunity to strengthen partnerships and broaden its impact. Leveraging GOARN’s well established mechanism for field deployment will help KDCA achieve its aspiration to make meaningful contributions to global response operations.
